# Comparison of muscle metabolomics between two Chinese horse breeds

**DOI:** 10.3389/fvets.2023.1162953

**Published:** 2023-05-05

**Authors:** Sihan Meng, Yanli Zhang, Shipeng Lv, Zhengkai Zhang, Xuexue Liu, Lin Jiang

**Affiliations:** ^1^Laboratory of Animal (Poultry) Genetics Breeding and Reproduction, Ministry of Agriculture, Institute of Animal Science, Chinese Academy of Agricultural Sciences (CAAS), Beijing, China; ^2^College of Animal Science, Xinjiang Agricultural University, Urumqi, China; ^3^CAAS-ILRI Joint Laboratory on Livestock and Forage Genetic Resources, Institute of Animal Science, Chinese Academy of Agricultural Sciences (CAAS), Beijing, China; ^4^Centre d'Anthropobiologie et de Génomique de Toulouse, Université Paul Sabatier, Toulouse, France

**Keywords:** horse (*Equus caballus*), untargeted metabolomics, muscle metabolism, lipid, glutathione metabolism

## Abstract

With their enormous muscle mass and athletic ability, horses are well-positioned as model organisms for understanding muscle metabolism. There are two different types of horse breeds—Guanzhong (GZ) horses, an athletic breed with a larger body height (~148.7 cm), and the Ningqiang pony (NQ) horses, a lower height breed generally used for ornamental purposes—both inhabited in the same region of China with obvious differences in muscle content. The main objective of this study was to evaluate the breed-specific mechanisms controlling muscle metabolism. In this study, we observed muscle glycogen, enzyme activities, and LC–MS/MS untargeted metabolomics in the gluteus medius muscle of six, each of GZ and NQ horses, to explore differentiated metabolites that are related to the development of two muscles. As expected, the glycogen content, citrate synthase, and hexokinase activity of muscle were significantly higher in GZ horses. To alleviate the false positive rate, we used both MS1 and MS2 ions for metabolite classification and differential analysis. As a result, a total of 51,535 MS1 and 541 MS2 metabolites were identified, and these metabolites can separate these two groups from each other. Notably, 40% of these metabolites were clustered into lipids and lipid-like molecules. Furthermore, 13 significant metabolites were differentially detected between GZ and NQ horses (fold change [FC] value ≥ 2, variable important in projection value ≥1, and *Q* value ≤ 0.05). They are primarily clustered into glutathione metabolism (GSH, *p* = 0.01), taurine, and hypotaurine metabolism (*p* < 0.05) pathways. Seven of the 13 metabolites were also found in thoroughbred racing horses, suggesting that metabolites related to antioxidants, amino acids, and lipids played a key role in the development of skeleton muscle in horses. Those metabolites related to muscle development shed a light on racing horses' routine maintenance and improvement of athletic performance.

## 1. Introduction

The excellent locomotion and racing performance of horses depend on their skeletal muscle metabolism. Therefore, the horse is a supremely effective animal model for understanding the effects of metabolism on muscle development (1). However, metabolite regulation mechanism associated with the training or development of horses remains unclear (2, 3). Previous studies have focused on diseases-related and exercise-related metabolites, for instance, the hematometabolic characteristics of blood metabolism in horses with atypical myopathy (AM) (4) and the identification of the biomarkers for acute laminitis by serum (5) metabolomics, and provide indexes for clinical diagnosis. Furthermore, the metabolomics of blood (6, 7) and muscle (8) for horses during endurance exercise reasons out the energetic demands for locomotion. Undoubtedly, metabolomics analysis has practical use for tracking stimulants from equine urine (9–11), and the analysis can also reflect exercise fatigue (12, 13) during exercise and training. Nevertheless, its utility is being increasingly acknowledged between different species [e.g., dogs (14, 15), chicken (16, 17), and cattle (18)]. Moreover, metabolomics has been applied to other livestock studies (19–21). Thus, metabolomics can afford opportunities to understand metabolic mechanisms between different breeds.

Many factors will affect muscle metabolism, such as exercise (8, 22), climate (23), and feed ingredients (24). To understand the muscle development related to breeds, we focused on two different horse breeds living in the Shaanxi Province of China that share similar herding management but exhibit different phenotypes (25). One is Guanzhong (GZ) horses, which have the characteristics of strong adaptability, tall body, easy feeding, fast growth and development, strong resistance to stress, and high reproduction rate (26), and the other one is the Ningqiang pony (NQ) horses, for which the average adult wither height is <106 cm as documented in a previous study (27). It adapts to the mountain areas with agile and small body size to carry heavy goods (28, 29). The variation in location between these two breeds requires muscle to provide power. Since the larger the muscle, the greater is its potential force output, skeletal muscle mass is important for a horse's performance and can vary greatly from breed to breed (30, 31). The huge difference in body size leads to different physiological changes in muscle composition and metabolism. There has been evidence that different breeds significantly affect muscle fiber structures, and regarding physiological changes, metabolism probably plays a key role during muscle formation (31).

Untargeted metabolomics is a comprehensive study that describes the entire endogenous metabolites in various organisms or tissues through the identification of metabolites in search of differential metabolites (32). The untargeted metabolomics can reflect the overall metabolite level and has been widely used to reveal the dynamic characteristics of metabolites under different conditions (33). As an approach to untargeted metabolomics, liquid chromatography–mass spectrometry (LC-MS) is considered to be the most stable and suitable technique for studying metabolism due to its wide coverage of substances (34). In the process of identifying metabolites, untargeted metabolomics may produce many false positive signals, when using the fragment ion of MS1 (35), whereas when combined with MS2, this bias can be alleviated. In this study, both MS1 and MS2 were used for the classification of metabolites and in the analysis; it was further enriched by the pathway, as reported in a previous study (36).

In the present study, we aimed to evaluate the breed-specific mechanisms related to horse muscle development. Thus, we performed biochemistry index detection and untargeted metabolomics analysis of muscle tissue from GZ and NQ horses. After the identification of metabolites, the significantly different metabolites between Ningqiang pony and Guanzhong pony muscles were analyzed. Our study would help understand the key components of metabolic networks and interactions during muscle formation. Elucidation of the underlying mechanisms contributes to the processes of feeding and exercise.

## 2. Materials and methods

### 2.1. Ethics statement

All animal experiments were conducted according to the regulations and guidelines established by the Animal Care Committee of the Institute of Animal Sciences, Chinese Academy of Agricultural Sciences (approval number: IAS2019-24). All incisions for muscle collection were performed under local anesthesia using 1% pentobarbital sodium, which is mainly used as an anesthetic agent for animals. Every effort was made to reduce animal suffering.

### 2.2. Sample collection by muscle biopsies

A total of 12 muscle samples were collected from Guanzhong horse (*n* = 6; male; age, 5.50 ± 2.42 years) and Ningqiang pony breeding center (*n* = 6; male; age, 4.89 ± 2.81 years), which are located in Shaanxi province, China (Supplementary Figure 1A). These two breeding centers share similar feeding and racing management, since they are near to each other (29). The feed ingredients are listed in Supplementary Table 1. Horses were weighed and examined by a clinician in a calm and quiet state prior to the muscle biopsy.

After preoperative disinfection with alcohol and iodophor, horses were subjected to local anesthesia (1 ml 1% pentobarbital sodium) in the gluteus medius (Supplementary Figure 1B). An appropriate amount of gluteus medius muscle was taken using an 8-mm biopsy punch (ACU-Punch^®^, Acuderm, Inc., Ft. Lauderdale, USA). The sample was placed into normal saline (0.9%NaCl solution) (Zhonghai Biotech, Beijing, China) to wash the blood, and tissues such as fat and fascia were removed. The final specimen was first stored in liquid nitrogen and then stored at −80°C until the metabolomic analysis. After sampling, we disinfected the wound with iodophor. For better postoperative recovery, Yunnan Baiyao powder was sprinkled over the incision until the dosage was sufficient to cover the wound (Yunnan Baiyao Group Co., Ltd, Kunming, China). Yunnan Baiyao [YNBY] is a traditional Chinese herbal medicinal formula widely used for its hemostatic effects and has been approved by the China Food and Drug Administration [CFDA] (37).

### 2.3. Detecting enzyme activity and muscle glycogen in the horse muscle

The glycogen content (nmol/L), hexokinase (HK) (mU/L) activity, and citrate synthase (CS) (U/L) activity of muscle were measured using the ELISA Kit (MLBio^®^, Shanghai, China) following their guidelines (38, 39). The substance extracted by the kit was determined under the condition of a 450-nm microplate reader.

### 2.4. Liquid chromatography mass spectrometry (LC–MS) measurement of metabolites

Metabolome measurements were performed by Lianchuan Biotechnology Co., Ltd (Hangzhou, China). First, a muscle biopsy was carried out to retrieve approximately 100-mg specimens, which were then ground and placed in liquid nitrogen. Then, the metabolites in the sample were extracted by shaking after adding 120 μl of 50% methanol (LC–MS grade, Honeywell^®^, Charlotte, USA) and treated at −20°C overnight to allow the proteins precipitated in the sample. After centrifugation (Heraeus™ Fresco17, Thermo Fisher Scientific^®^, Waltham, USA), the supernatants were transferred into a 96-well plate. To confirm its stability and repeatability, 10 μl was extracted from each sample and prepared as a quality control (QC) sample.

Compounds were analyzed in positive and negative ion modes by the TripleTOF 5600+ high-resolution tandem mass spectrometer (SCIEX^®^, Warrington, UK). Chromatographic separation was performed using an ultraperformance liquid chromatography (UPLC) system (SCIEX^®^, UK) with default parameters. The ACQUITY UPLC T3 column (100 mm × 2.1 mm, 1.8 μm, Waters, UK) was used for reverse-phase separation. Solvent A (water, 0.1% formic acid) and solvent B (acetonitrile, 0.1% formic acid) were used as the mobile phase to separate metabolites. A high-resolution mass spectrometer, TripleTOF 5600+ (SCIEX, UK) time-of-flight mass spectrometry was selected for the detection of eluted metabolites in the column. The ion spray floating voltage is set to 5 kV in positive ion mode and −4.5 kV in negative ion mode. The mass spectroscopy (MS) data were collected using Information Dependent Acquisition (IDA) mode. The mass range of TOF is 60–1,200 Da and survey scans are acquired every 150 ms. The first 12 signal ions with a positive charge (or a negative charge in the negative ion mode) and signal accumulation intensity of more than 100 times/s were selected from the MS1 map for MS2 fragmentation scanning. At the same time, every 10 QC samples were scanned. The quality difference between QC was used to correct the systematic error of the entire batch experiments.

### 2.5. Metabolomics data proceeding

**Figure 1:**
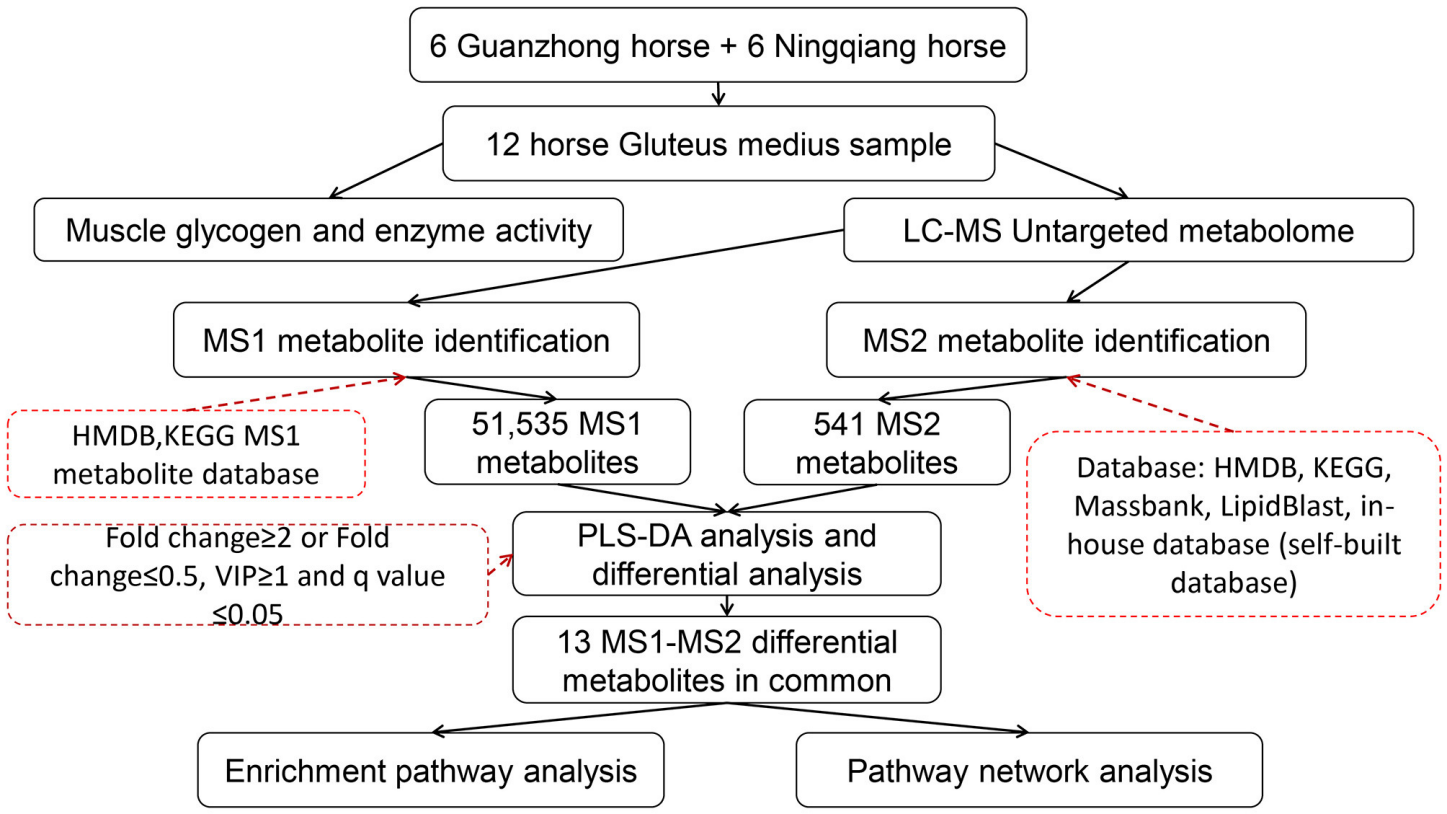
Study design.

The raw data were first processed using XCMS (40), which took time–*m*/*z* pairs, retention time, and peak area into consideration. Then, they were extracted and identified by msConvert (41) of the Proteowizard (42), XCMS (40), CAMERA (43), and metaX (44) packages that were included in R (v.4.1.2). The Total Ion Chromatogram (TIC) diagram utilizes retention time as the abscissa and the total intensity of each ion at each time point in the mass spectrum as the ordinate. The TIC peak retention time and peak area of the QC samples in this study overlapped well, indicating that the instrument was stable and reliable (Supplementary Figure 2). The *m*/*z* values of metabolites mostly range from 200 to 800 and the retention time mostly ranges from 0 to 4 min. The main workflow design of the study is shown in Figure 1.

For the identification of metabolites, we relied on three criteria: retention time (RT), accurate mass match to the library ± 10 ppm (MS1), MS/MS spectra (MS2) with high forward and reverse scores, as reported in previous studies (45–47). MS1 compounds were identified using the database of The Human Metabolome Database (HMDB) (www.hmdb.ca) (48) and Kyoto Encyclopedia of Genes and Genomes (KEGG) (https://www.kegg.jp/) (49), while MS2 metabolites were further identified using multiple databases (HMDB, KEGG; Massbank (http://www.massbank.jp/) (50); LipidBlast (51); and in-house database) as shown in Figure 1. Among them, the in-house database (self-built database) contains MS2 spectra of about 20,000 kinds of standard products (positive ions: 66,124; negative ions: 20,088). Due to certain differences in the species and quantities of metabolites between the two modes (Supplementary Figure 3), taken MS1 and MS2 together can alleviate the false positive rate to a large extent. We annotated these metabolites using CAMERA (43) and metaX (44). Specific parameters of the software are provided in Supplementary Tables 2–4.

### 2.6. Quality control analysis (QC)

Features with missing rate—more than 50% in QC samples or 80% in test samples—were removed, and the values for missing peaks were extrapolated with the k-nearest neighbor algorithm to further improve the data quality. In addition, the relative standard deviations of the metabolic features were calculated across all QC samples, and those with standard deviations >30% were removed. Data normalization was performed on all samples using the probabilistic quotient normalization algorithm. Then, QC-robust spline batch correction was performed using QC samples. Principal component analysis (PCA) was performed to detect outliers and batch effects using the pre-processed dataset.

### 2.7. Statistical analysis

Student's t-tests were used to identify metabolites that differed significantly between GZ and NQ horses. Two estimates of the false discovery rate (FDR, *q*-value) and the Benjamin–Hochberg procedure were calculated to correct the *p*-values. We also conducted the supervised Partial least squares discriminant analysis (PLS-DA) using metaX (44) for variables that discriminated the profiling statistical method. To verify the PLA-DA results, the displacement tests of R2 and Q2 were carried out for 200 times. R2 is the goodness-of-fit parameter and Q2 is the predictive ability parameter, which are used to verify the accuracy and predictability of the PLS-DA model. Multivariate analysis and PLS-DA discriminant analysis were used to obtain the variable important in projection (VIP) value of each metabolite variable. Differentially expressed metabolites were screened using VIP values of the first two principal components of the multivariate PLS-DA model, combined with *p*-values and fold changes, following the criteria, namely, fold change of ≥ 2, VIP of ≥ 1, and *q*-value of ≤ 0.05. The result was showed by a volcano plot using the R (v.4.1.2) packages of “ggplot2”.

A mental test was performed to establish connections between phenotype indexes and metabolites using the R (v.4.1.2) packages of “ggcor.”. The Spearman correlation coefficient was used to analyze the correlations between metabolome and functional enzymes with a threshold of *p* of < 0.05. Heatmaps were used to visualize different patterns of those differential metabolites between GZ and NQ horses. The distance was calculated as the Euclidean distance, with the clustering method of “ward D2”. Similarly, a scatter bubble diagram was introduced to show the metabolic pathways from the result of MetPA and pathway analysis module of the MetaboAnalyst 5.0 online platform (52).

## 3. Results

### 3.1. The variability muscle glycogen concentration in GZ and NQ horses

**Figure 2:**
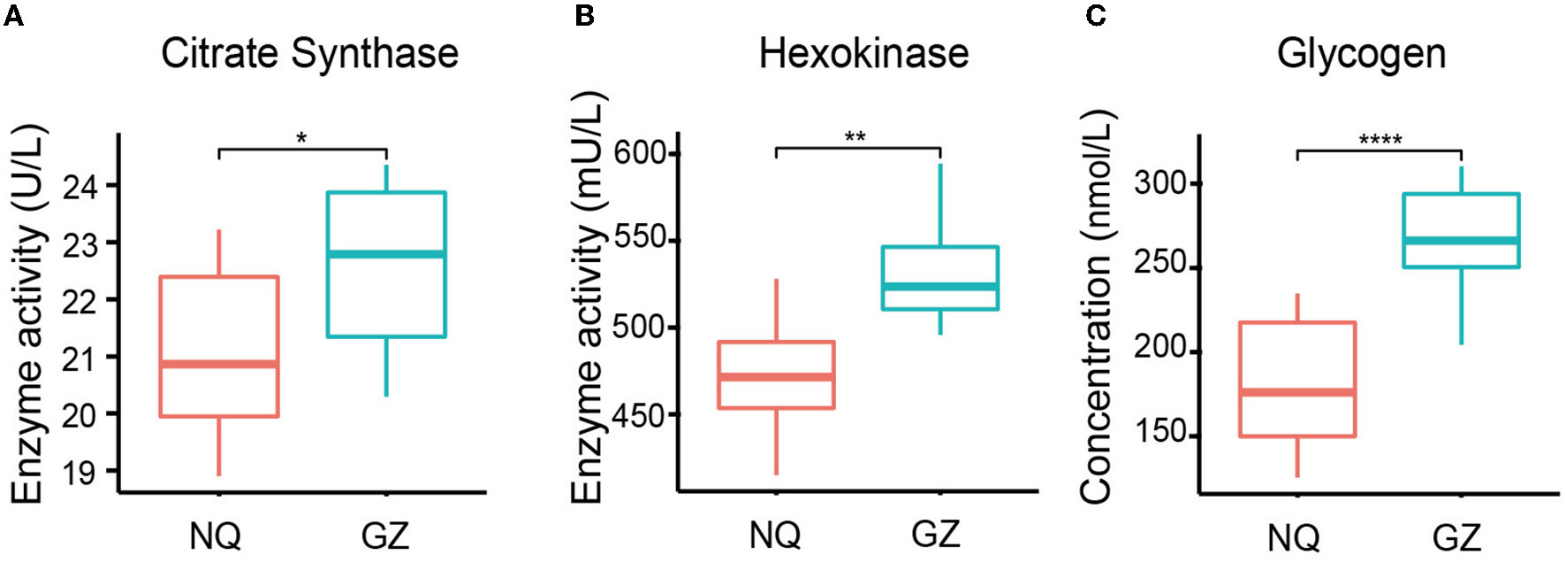
Muscle glycogen content and TCA oxidase activity of GZ and NQ horses. **(A)** The variability of citrate synthase activity in GZ and NQ horses. **(B)** The variability hexokinase activity in GZ and NQ horses. **(C)** The variability muscle glycogen concentration in GZ and NQ horses. ^*^: *p* ≤ 0.05; ^**^: *p* ≤ 0.01; ^***^: *p* ≤ 0.001; ^****^: *p* ≤ 0.0001.

We found that the glycogen concentration was significantly high in the muscles of GZ horses (*p* = 4.8e–6) (Figure 2C) and that the citrate synthase (*p* = 0.02) and hexokinase (*p* = 0.006) activity were also high in GZ horses (Figures 2A, B). The citrate synthase and hexokinase activity are the core enzymes of the TCA cycle. Mental analysis showed a significant correlation between the identified metabolites and the muscle glycogen content (Supplementary Figure 4).

### 3.2. Metabolite detection and classification using MS1 and MS2

**Figure 3:**
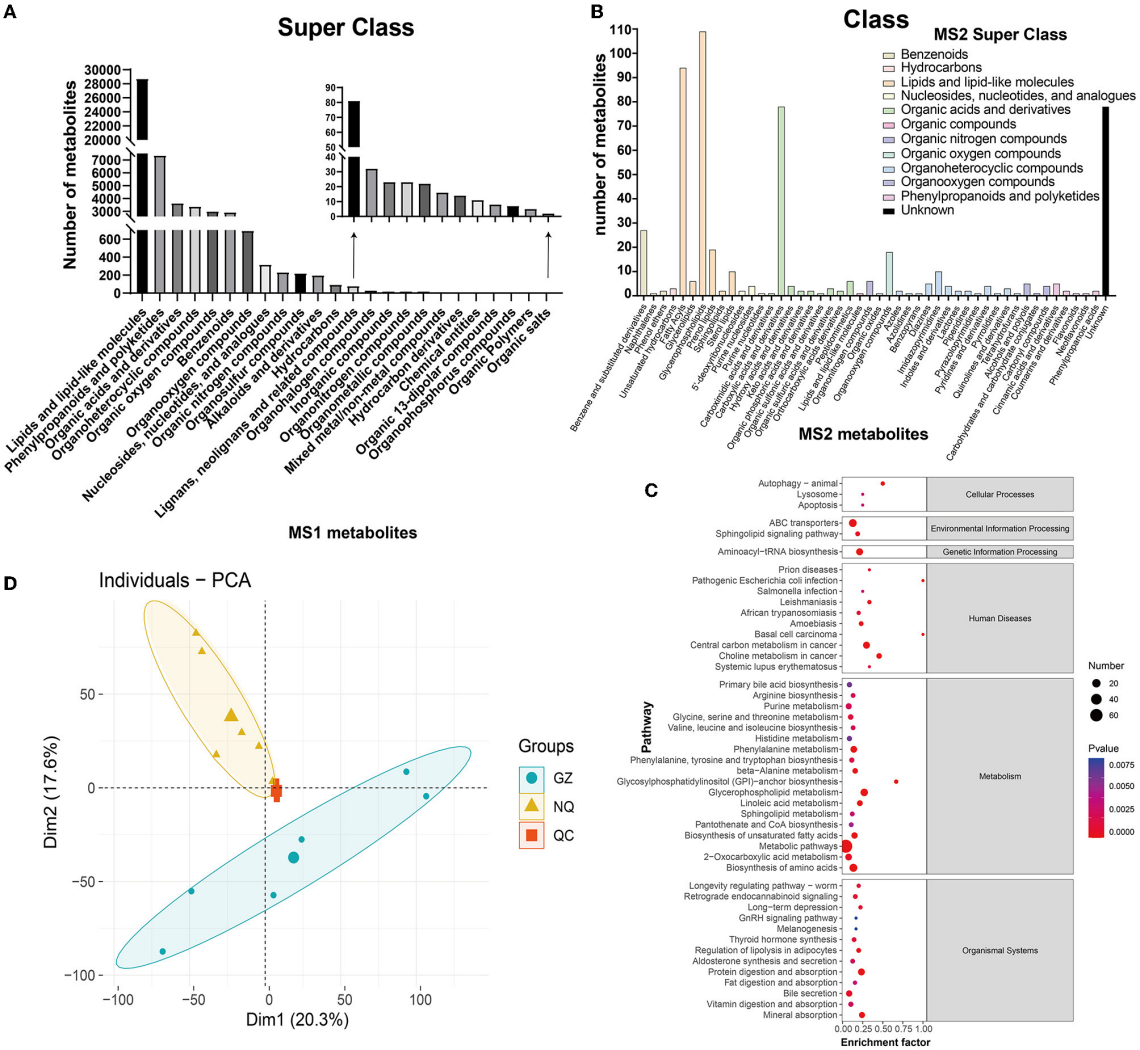
**(A)** MS1 metabolite classification bar chart. **(B)** MS2 metabolite classification bar chart. The abscissa represents the species of MS2 metabolites annotated to the class. Different colors in the bar chart represent different superclass categories. **(C)** A bubble map of MS2 metabolite pathway. The bubble color represents the size of *p*-value, and the smaller the *p*-value, the redder the color, the larger the *p*-value, and the bluer the color. Bubble size represents the amount of MS2 metabolites enriched. **(D)** PCA plot. The blue circle represents the Guanzhong (GZ) horse muscle individual, the yellow triangle represents the Ningqiang pony (NQ) horse muscle individual, and the red square represents the quality control (QC) individual. Each scatter represents a sample.

A total of 51,535 metabolites were annotated by the HMDB database, which included 30,539 MS1 metabolites in positive mode, and included 20,996 in negative mode (Supplementary Table 5). All these metabolites can be classified into 24 superclass categories, and the first six groups accounted for more than 95% of all these metabolites; they were listed as lipids and lipid-like molecules (55.79%); phenylpropanoids and polyketides (14.28%); organic acids and derivatives (7.08%); organoheterocyclic compounds (6.58%); organic oxygenated compounds (5.86%); and benzenoids (5.72%). It is worth mentioning that these six categories accounted for more than 95% of all these metabolites (Figure 3A and Supplementary Figure 5). A total of 15,978 out of 51,535 MS1 metabolites were annotated into 40 pathways and 6 categories according to the KEGG database. Similarly, metabolites were most abundant in the amino acids and lipid metabolism pathways (Supplementary Figure 6 and Supplementary Table 5).

Additionally, these different substances were fragmented and were used to generate the MS2 mass spectrogram, according to the standard substance from the database. Then, they were scored to yield the matching result that ultimately provides the MS2 identification results of the metabolites (Supplementary Table 4). As a result, a total of 374 metabolites defined by MS2 were detected in positive mode and 167 in negative mode (Supplementary Table 5). Interestingly, nearly 44% of metabolites were clustered into lipids and lipid-like molecules in the superclass classification of these MS2 metabolites. Further annotation showed that they were clustered into glycerophospholipids (~20%), organic acids and derivatives (~18%), fatty acyls (~17%), and carboxylic acids and derivatives (~14%), as well as unknown (31%), and the detailed list is shown in Figure 3B and Supplementary Tables 6, 7. When these MS2 metabolites were enriched by the KEGG database, they were annotated into 47 pathways and 6 major groups (Figure 3C). Most of them were enriched in the metabolism pathway, which is similar to the result identified by MS1.

Both metabolites identified by MS1 and MS2 models were used for PCA. The results showed that GZ and NQ horses could be differentiated clearly and that PCA1 and PCA2 could explain 37.9% of metabolite variation between the two populations (Figure 3D). Reproducible aggregation of QC samples indicates good stability of the instrument and high quality of the measurement data.

### 3.3. Different muscle metabolites identified between GZ and NQ horses

**Figure 4:**
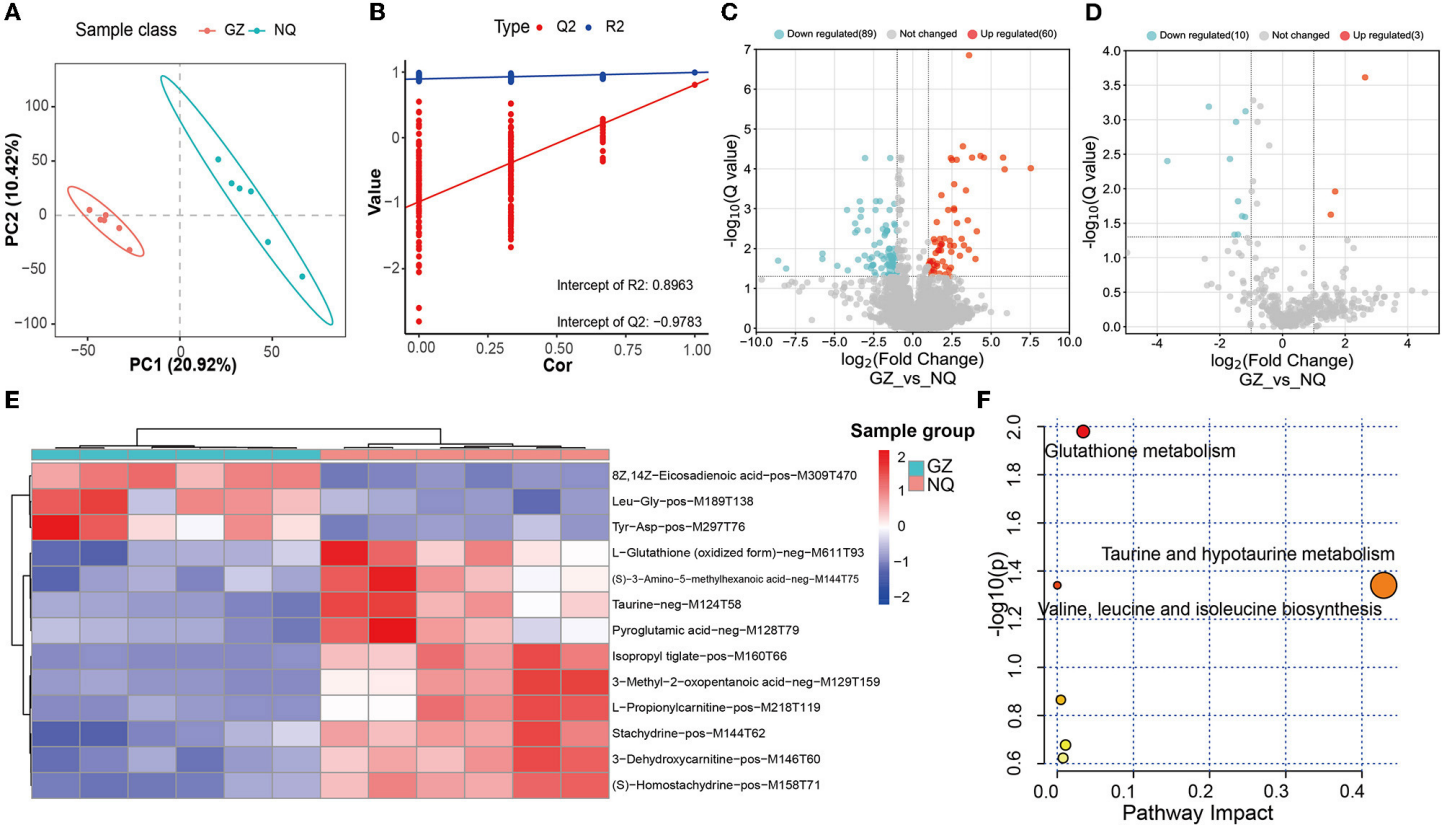
**(A)** PLS-DA score plot. Red represents the Guanzhong horse muscle group (GZ), blue represents the Ningqiang pony horse muscle group (NQ). **(B)** PLS-DA permutation test diagram showing the plots of R2, Q2, and accuracy. **(C)** A volcano plot of MS1 differential metabolites. Red represents upregulated metabolites and blue represents downregulated metabolites; *Q* = 0.05 was used as the threshold of whether the metabolites were significantly different. **(D**) A volcano plot of MS2 differential metabolites. **(E)** Cluster analysis of 13 differential. Six samples were Guanzhong (GZ) in blue group, and six samples were Ningqiang (NQ) in red group. **(F)** MetPA analysis of 13 differential MS2 metabolites pathway. The larger the bubble and abscissa values, the more important the path. The red color indicates the high impact of the respective metabolic pathway. *p* < 0.05 (significant).

The PLS-DA score plot showed that GZ and NQ horses could be separated at PC1 with a variation of ~20%, which mirrors the result of PCA (Figure 4A). In the permutation test plot of the PLS-DA model, the results showed that R2 and Q2 both decreased gradually with the gradual decrease in replacement retention, proving appropriate robustness of the model, and most of the Q2 values <0 indicated that the analysis of differential metabolites was accurate (Figure 4B). Of the MS1 metabolites identified in the muscle biopsy samples, only 149 were significantly altered between GZ and NQ horses (60 upregulation; 89 downregulation; *Q* < 0.05; FC > 2, VIP ≥ 1) (Figure 4C).

Furthermore, by using the same strategy, 13 significant metabolites were identified, including 3 upregulated and 10 downregulated metabolites (Figure 4D). We found that there are 13 common differential metabolites from both MS1 and MS2 (Supplementary Table 8). Moreover, these 13 MS2 metabolites were classified into three superclass and five class categories (Supplementary Table 10).

### 3.4. Glutathione metabolism and taurine metabolism alter between GZ and NQ horses

With these abovementioned metabolites, we then made a cluster analysis to explore functional components indicative of muscle development (Figure 4E). In this way, a total of five metabolic pathways were detected, including glutathione metabolism (*p* = 0.01) and taurine and hypotaurine metabolism pathways (*p* = 0.04) (Figure 4F and Supplementary Table 9), suggesting that antioxidant supplementation responds to oxidative stress during muscle development.

### 3.5. Muscle metabolic network inferred for GZ and NQ horses

**Figure 5:**
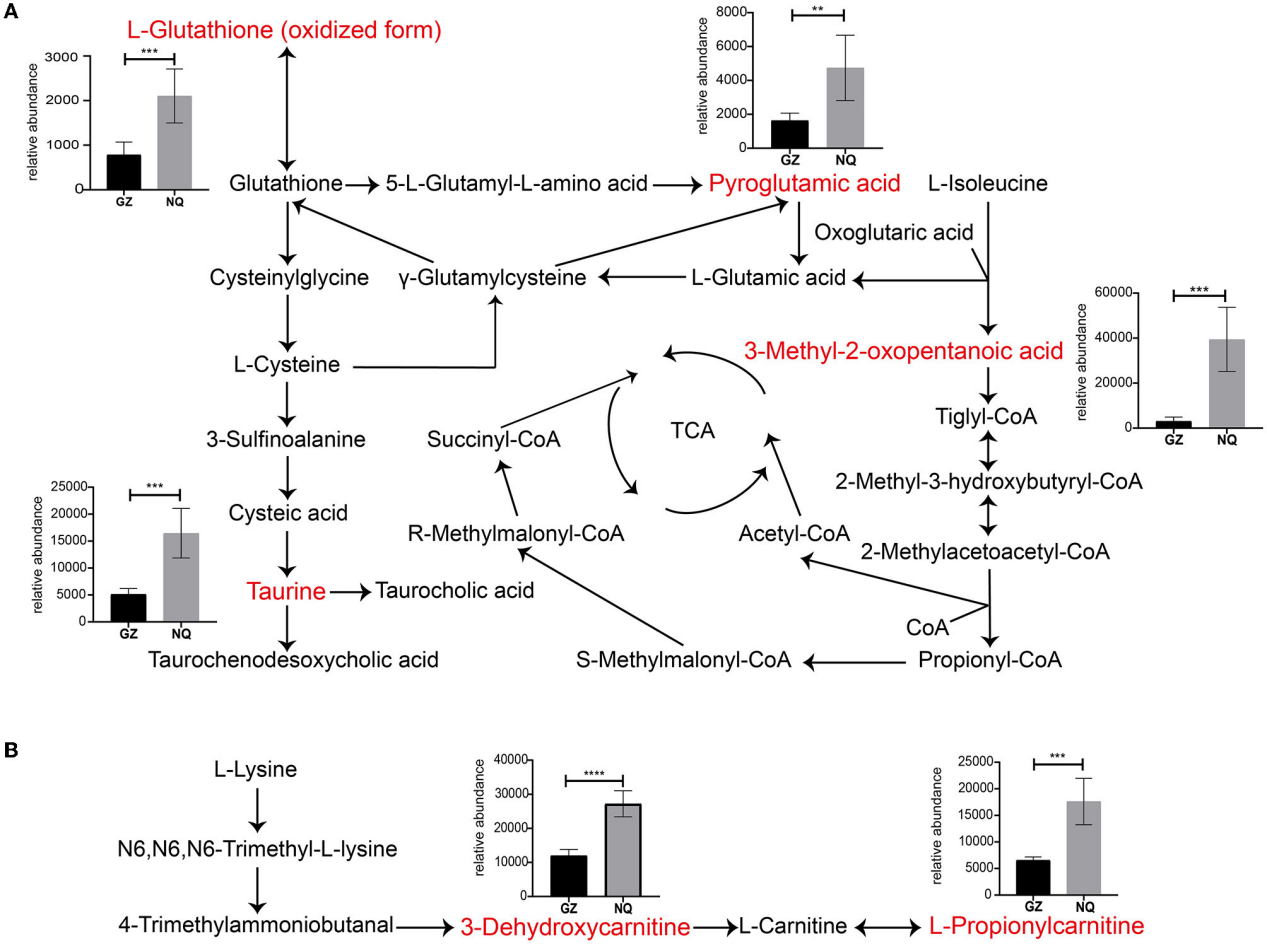
**(A, B)** Thirteen significantly different metabolites pathway network graphs. Red color indicates the differential metabolites analyzed. The ordinate of the bar represents the relative abundance equivalent to the metabolite content. GZ, Guanzhong horse; NQ, Ningqiang pony horse. ^*^: *p* ≤ 0.05; ^**^: *p* ≤ 0.01; ^***^: *p* ≤ 0.001; ^****^: *p* ≤ 0.0001.

For a clear understanding of the interactive role of metabolites that contribute to muscle development, we demonstrate a metabolic network diagram to visualize the result in Supplementary Figure 7. As shown in Figures 5A, B, these metabolites are not independent but act as hub roles in the metabolite networks. For instance, the glutathione (GSH) metabolism pathway regulates taurine (TU), the hypotaurine (HTU) metabolism pathways regulates antioxidative mechanisms, and bile acid biosynthesis regulates GSH generation to modulate the oxidative response. The valine, leucine, and isoleucine degradation pathways are involved in the tricarboxylic acid cycle (TCA). Among them, 3-methyl-2-oxopentanoic acid can regulate the TCA cycle by producing tiglyl-CoA (Figure 5A). As intermediate metabolites, L-hydroxycarnitine and 3-hydroxycarnitine metabolites serve important roles in carnitine synthesis and oxidation of branched-chain fatty acid pathways (Figure 5B). These key metabolites were significantly more abundant in NQ horses than in GZ horses (Figures 5A, B).

## 4. Discussion

With their large skeletal muscle mass and innate ability to exercise, horses are positioned as a unique model organism, and they can be used to understand the effects of breed differences, specifically on muscle metabolism. As one of the largest skeletal muscle of horses, the gluteal muscle has a function in stabilizing the limb during stance and locomotion (53). This study shows the presence of higher glycogen concentration in GZ horses than in NQ horses, which is the major source of energy during muscle development (54); the higher level of glycogen reflects stronger athletic performance in GZ horses. Besides, citrate synthase and hexokinase activity that played key roles in the TCA cycle (Figures 2A, B) were also associated with glycogen utilization to produce more energy for muscle growth and racing activities. These measurements may provide meaningful markers for the prediction of the strength of horse muscle locomotion ability. We then found metabolites in GZ and NQ horses, which were clustered into lipids and lipid-like molecules; these components are related to energy storage and production. These findings demonstrate that glycolytic and energy-related metabolisms have better performance in the racing breed horses to store and utilize more energy than short-stature horses (55).

There were 13 MS2 significantly differential metabolites detected in this study between the NQ and GZ horse breeds (Figure 4D); among them, L-glutathione (oxidized form), pyroglutamic acid, taurine, 3-dehydroxycarnitine, and L-propionylcarnitine metabolites were significantly associated with muscle movement, as reported in previous studies (56–60). Seven of these metabolites can be found in exercise training horses: taurine, L-propionylcarnitine, 3-dehydroxycarnitine (deoxycarnitine), 3-methyl-2-oxopentanoic acid (3-methyl-2-oxovalerate), Leu-Gly (L-leucylglycine), L-glutathione (oxidized form), and pyroglutamic acid (5-oxoproline) (58, 59, 61, 62); this indicates that some “housekeeping” ingredients functioned well during this process. Notably, three of them are related to the lipids and lipid-like molecules. Carnitine is essential for the transport of activated fatty acids across the mitochondrial membrane during mitochondrial β-oxidation (63). Muscles take up carnitine from blood in an exchange–diffusion process with endogenous deoxycarnitine, the immediate precursor of carnitine (64), and L-propionylcarnitine is an acylcarnitine. The general role of acylcarnitines is to transport acyl groups (organic acids and fatty acids) from the cytoplasm into the mitochondria so that they can be broken down to produce energy. This process is known as β-oxidation. L-Propionylcarnitine is a carnitine derivative that has a high affinity for muscular carnitine transferase: it increases cellular carnitine content, thereby allowing free fatty acid transport into the mitochondria. Long-term oral treatment with propionyl-L-carnitine improves maximum exercise duration and maximum oxygen consumption over placebo and indicates a specific propionyl-L-carnitine effect on peripheral muscle metabolism (65, 66). All of these indications suggest that lipid metabolism play an important role in muscle development and provides energy during this process (12). This may contribute to the differentiation of muscle development between GZ and NQ horses.

Further analysis demonstrated that glutathione metabolism (*p* = 0.01; Figure 4F) and the taurine and hypotaurine metabolism pathways (*p* = 0.04; Figure 4F) were significantly associated with muscle metabolic mechanisms. Moreover, these metabolites interact with each other and treated as rate-limiting metabolites in the TCA cycle, which generate energy for biological process. The glutathione metabolism pathway is of important metabolite for antioxygen. The L-glutathione (oxidized form) is a ubiquitous and powerful cellular antioxidant essential for protecting cells from the harmful effects of oxidative damage and free radical damage. It is a highly abundant metabolite in cells (67). Furthermore, the taurine pathway, which is widely distributed in animal tissues and has many biological functions, such as an important role in osmotic pressure regulation, act as a stabilizer for the cell membrane and protein, having antioxidant and anti-inflammatory functions, regulating mitochondrial tRNA activity, and participating in calcium homeostasis (68–70). Its deficiency leads to muscle dysfunction and affects athletic performance (70–72), and is related to skeletal muscle aging (73, 74). This is reasonable along with the notion that the muscle glycogen content is significantly the Pearson association with those metabolites (Supplementary Figure 4) because the more the energy resource, the more is the metabolites produced. These significant metabolites reveal differences in glycolytic metabolism and exercise capacity in horse muscles of different breeds. It provided a theoretical basis for the muscle metabolism analysis of different species and for the direction of identification and breeding.

## 5. Conclusion

In this study, the differential metabolites in muscle were detected between two different horse breeds (GZ and NQ) living in the same area. We found that the GZ athletic horses exhibit higher muscle glycogen contents and higher enzyme activities than NQ horses. While, notably, the lipid and lipid-like taurine and taurine hypotaurine metabolism and glutathione metabolism, which were interactive in the TCA cycle, were significantly decreased in GZ horses. This indicates that GZ horses possess higher energy storage and utilization ability to meet its requirement for exercise and racing. In summary, we conclude that these metabolites have the potential to be used as an indicator to understand the metabolic aspects of exercise capacity in different breeds of horses. In future, *in vivo* and *in vitro* validations will be needed to understand the biological mechanisms.

## Data availability statement

The original contributions presented in the study are included in the article/Supplementary material, further inquiries can be directed to the corresponding author.

## Ethics statement

The animal study was reviewed and approved by Animal Care Committee of the Institute of Animal Sciences, Chinese Academy of Agricultural Sciences (approval number: IAS2019-24).

## Author contributions

SM prepared samples, performed statistical analyses, and wrote the manuscript. YZ collected samples, prepared samples, and reviewed the manuscript. SL and ZZ collected samples. XL idealized the project and reviewed the manuscript. LJ and XL supervised this work. All authors contributed to and approved the final manuscript.
