# Supplementary material for: Comparison of muscle metabolomics between two Chinese horse breeds
Source: Front Vet Sci. 2023 May 5;10:1162953. doi: 10.3389/fvets.2023.1162953 (PMC10196265; doi:10.3389/fvets.2023.1162953)
Supplement: Supplementary file 5 [file Data_Sheet_5.PDF]

Supplementary Material

Supplementary_Material-Figure：PDF containing Supplementary Information

Figure S1. A) Geographic distribution of horses. **B)** sampling position; **Figure S2.** TIC (Total Ion Chromatogram) (pos and neg); **Figure S3.** Distribution map of metabolites m/z-rt (pos and neg); **Figure S4.** Mental analysis of muscle glycogen content and TCA core enzyme activity; **Figure S5.** HMDB Super class classification diagram of MS1 metabolites; **Figure S6.** KEGG pathway classification map of MS1 metabolites; **Figure S7.** Contents of 13 MS2 differential metabolites in muscle of two different breeds of horse. Guanzhong(GZ), Ningqiang(NQ). (PDF)

1 Supplementary Figures

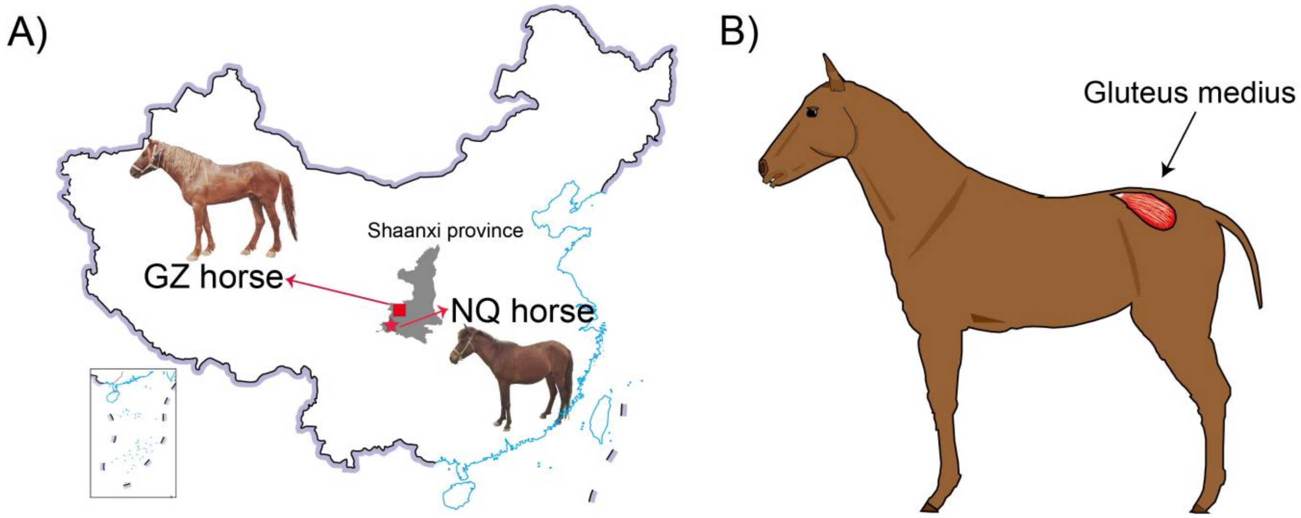

Figure S1. A) Geographic distribution of horses. **B)** sampling position

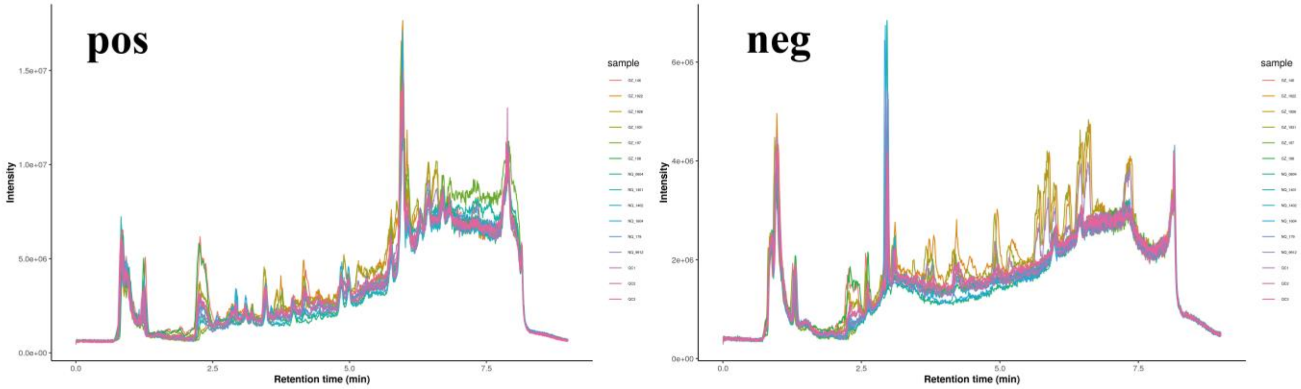

Figure S2. TIC（Total Ion Chromatogram）（pos and neg）. The Total Ion Chromatogram in positive Ion mode is shown on the left, and the Total Ion Chromatogram in negative Ion mode is shown on the right. The abscissa is the ion retention time, the ordinate is the total intensity of each ion at each time point in the mass spectrum, and each color represents a sample.

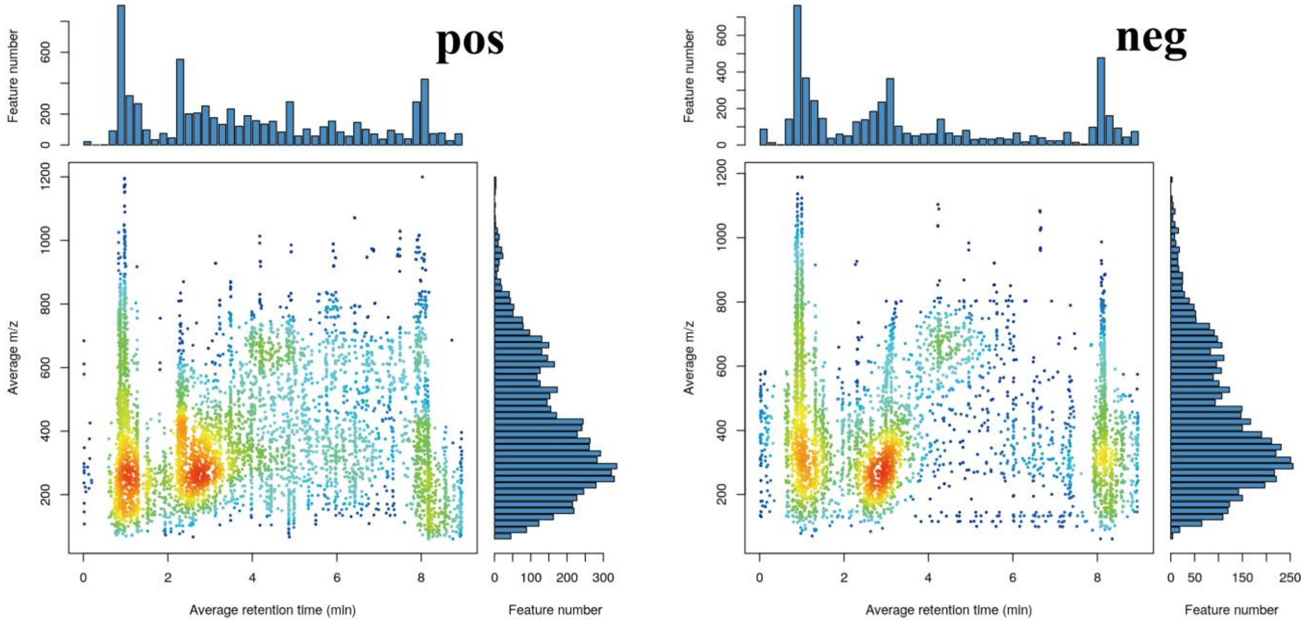

Figure S3. Distribution map of metabolites m/z-rt (pos and neg). Distribution map of metabolites m/z-rt (pos and neg). The left figure is in positive mode, and the right figure is in negative ion mode. The abscissa of the rectangle represents the average retention time of these ions in minutes. The ordinate of the rectangle represents the mass to charge ratio (m/z) of the average charged particle of these ions. The Feature number in the horizontal bar chart represents the number of ions with different average m/z; The Feature number on the longitudinal bar chart represents the number of ions with different average retention time. The dots in the rectangle represent ions.

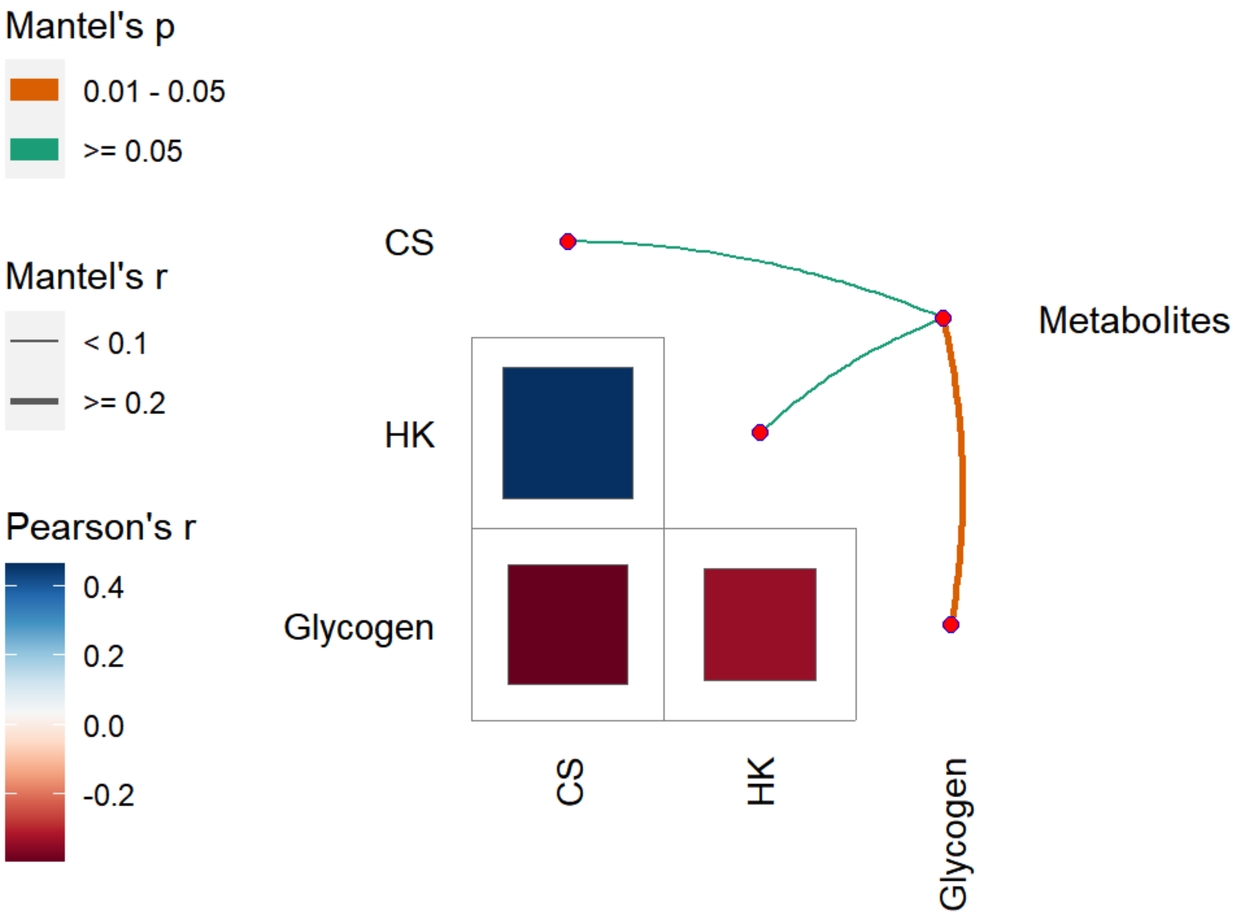

Figure S4. Mental analysis of muscle glycogen content and TCA core enzyme activity;

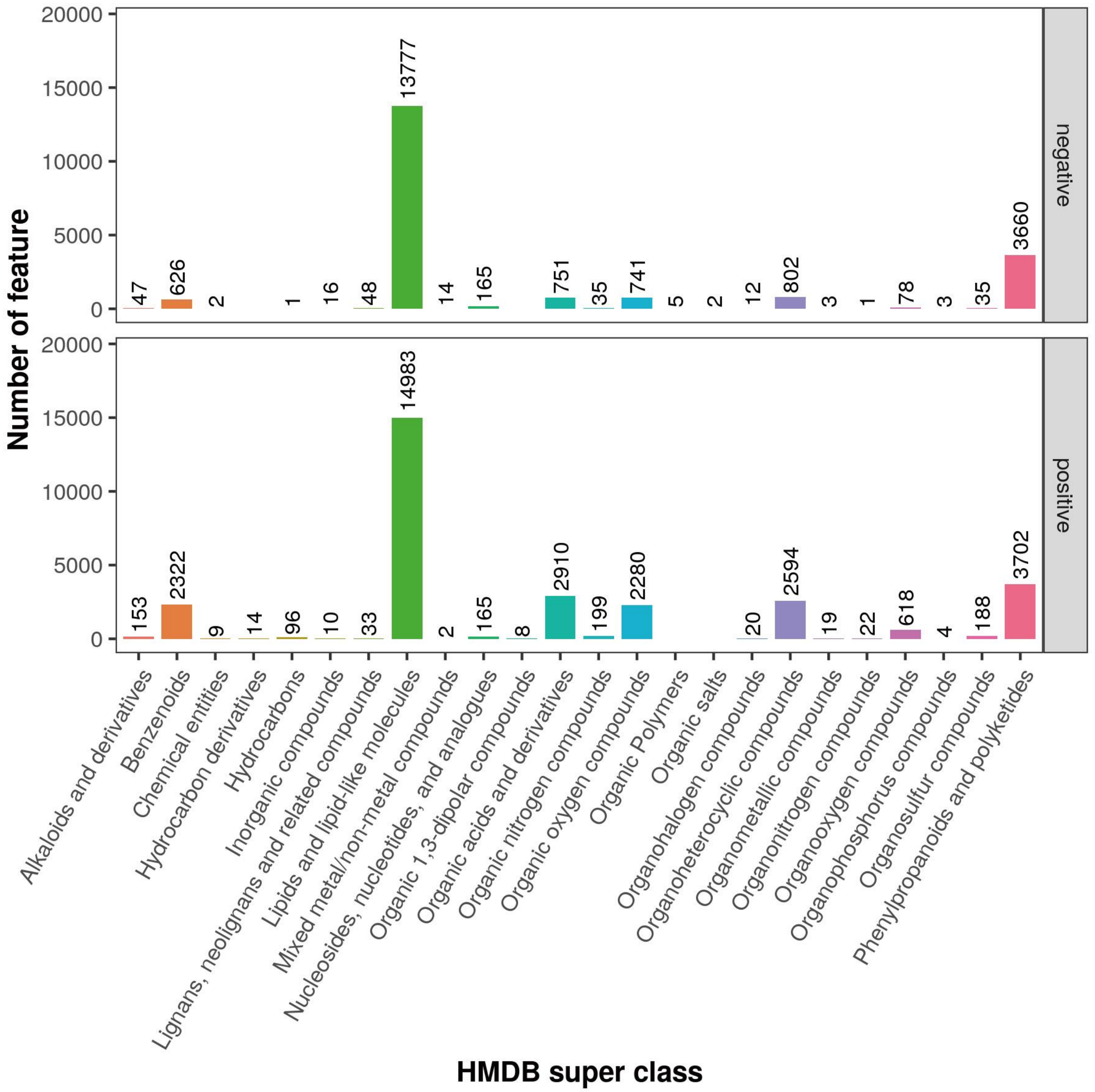

Figure S5. HMDB Super class classification diagram of MS1 metabolites. Different colors in the bar chart represent different super class species of MS1 metabolites.

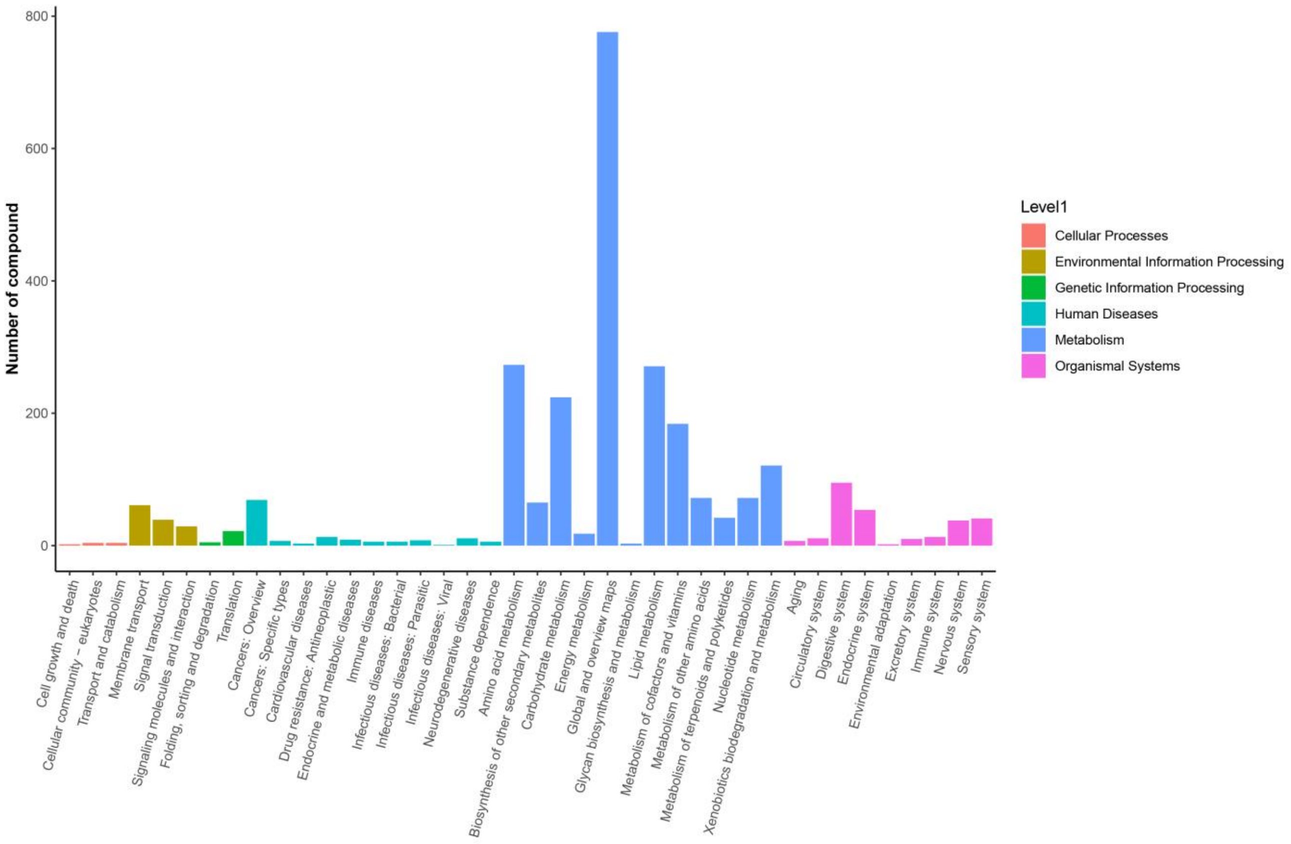

Figure S6. KEGG pathway classification map of MS1 metabolites. The abscissa represents level 2 metabolic pathways annotated by MS1 metabolites. Different colors in the bar chart represent different level 1 pathways.

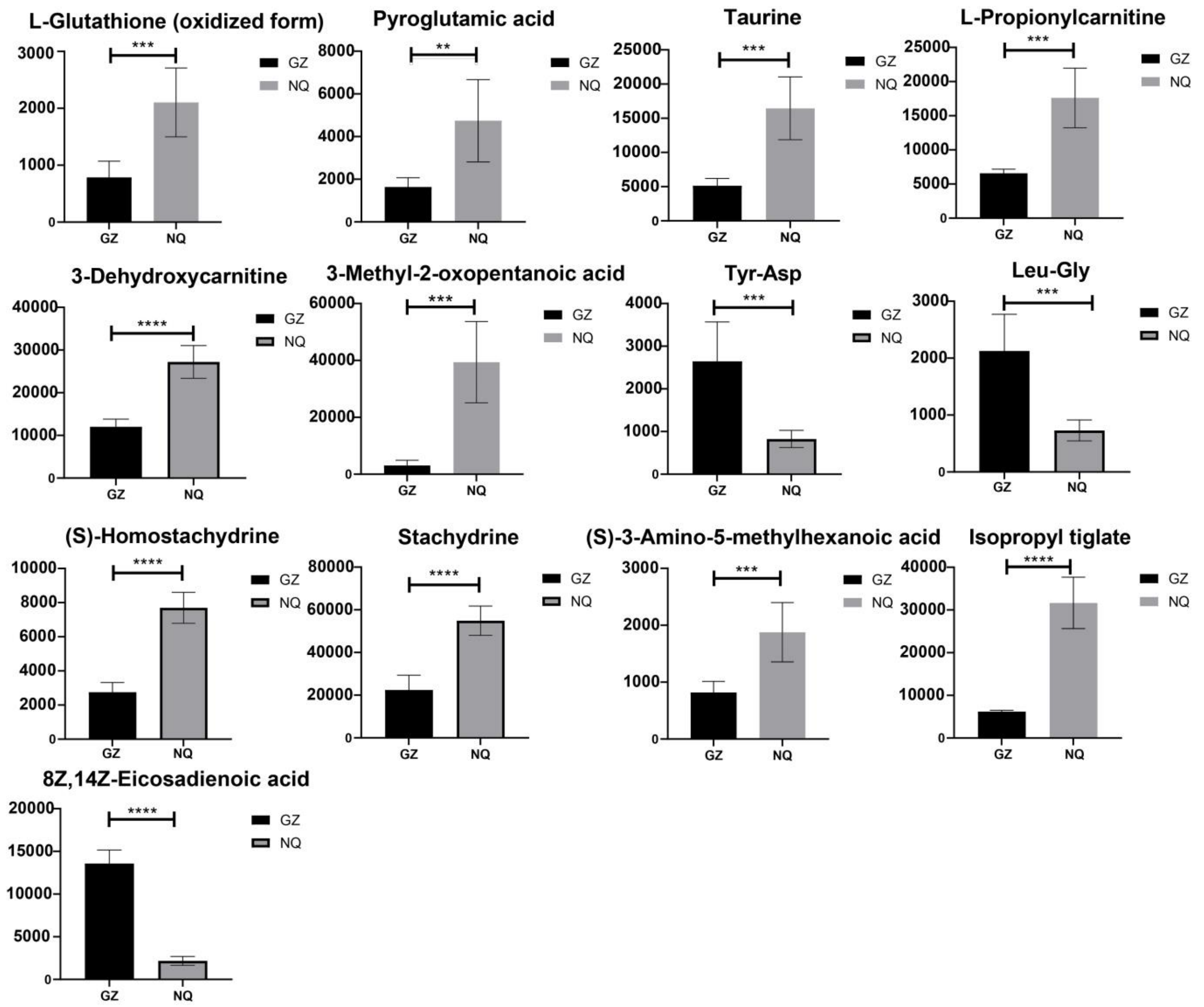

Figure S7. Contents of 13 MS2 differential metabolites in muscle of two different breeds of horse. Guanzhong(GZ), Ningqiang(NQ). Ordinate represents the value of peak area. Because the peak area is proportional to the content, it can be used to characterize the content of metabolites. *: $P \leq 0.05$; **: $P \leq 0.01$; ***: $P \leq 0.001$; ****: $P \leq 0.0001$.
